# 949. Impact of Antimicrobial Stewardship Pharmacist Participation in Pediatric Infectious Diseases Rounds on Prospective Audit and Feedback Recommendations

**DOI:** 10.1093/ofid/ofac492.792

**Published:** 2022-12-15

**Authors:** Laura Bio, Lauren M Puckett, Torsten Joerger, Hayden T Schwenk

**Affiliations:** Lucile Packard Children's Hospital Stanford, Palo Alto, California; Stanford Children's Hospital, Stanford, California; Stanford University School of Medicine, Stanford, California; Stanford University School of Medicine, Stanford, California

## Abstract

**Background:**

Prospective audit and feedback (PAF) is a core strategy of antimicrobial stewardship programs (ASPs). To improve communication of PAF recommendations to the inpatient pediatric infectious diseases (ID) consult service, the ASP pharmacists at our hospital were integrated into ID rounds. The purpose of this study was to assess the impact of ASP pharmacy participation in ID rounds on the rate of ASP recommendations.

**Methods:**

Prior to implementation of the ASP pharmacist rounding service, the ASP pharmacist did not routinely attend rounds or communicate PAF recommendations directly to the ID consult service. Starting 1/3/22, the ASP pharmacists had daily (M-F) in-person discussions with the ID team regarding their patients’ antimicrobials. Audits performed between 1/4/21-12/30/21 and 1/3/22-4/29/22 on patients with an ID consult were included in the non-rounding cohort (NRC) and rounding cohort (RC), respectively. We compared PAF recommendation rates, characteristics, and acceptance rates between the two cohorts.

**Results:**

There was an increase in PAF recommendation rate in the RC compared to NRC (188/485 [39%] vs 359/1234 [29%], p < 0.001). Antibiotics were the antimicrobial category mostly likely to have a recommendation and the rate of antibiotic PAF recommendations was higher in the RC compared to the NRC (132/341 [39%] vs. 271/934 [29%], p = 0.001) (Table 1). The most common recommendation types in both cohorts were to optimize the antimicrobial dose and antimicrobial discontinuation. Recommendations were more frequently communicated to the ID team in the RC compared to NRC (125/188 [66%] vs. 107/359 [30%], p < 0.001). The recommendation acceptance rate was similar between the two cohorts (159/188 [85%] RC vs. 290/359 [81%] NRC, p = 0.29).

**Figure:**
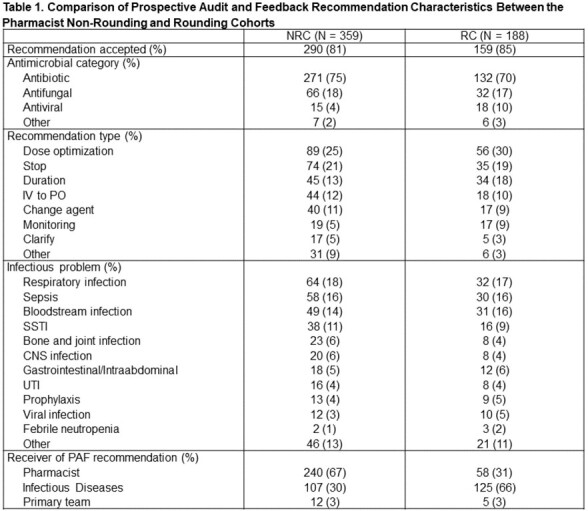

CNS: Central nervous system; IV: Intravenous; NRC: Non-rounding cohort; PAF: Prospective audit and feedback; PO: Per os, oral; RC: Rounding cohort; SSTI: Skin and soft tissue infection; UTI: Urinary tract infection.

**Conclusion:**

Implementation of a pediatric ASP pharmacist rounding service increased the PAF recommendation rate and improved recommendation communication with the pediatric ID consult service. Participation in rounds may better inform ASP pharmacist PAF recommendations. Future studies describing the potential benefit to the ID team by having an ASP pharmacist present on rounds are warranted. ASPs should consider formal integration of ASP pharmacists as part of the ID consult service to further improve the quality of antimicrobial prescribing.

**Disclosures:**

**All Authors**: No reported disclosures.

